# Assessing the reproducibility and predictive value of objective cough measurement for successful withdrawal of invasive ventilatory support in adult patients

**DOI:** 10.1186/s12890-024-03033-6

**Published:** 2024-05-02

**Authors:** Fabio Varón-Vega, Adriana Rincón, Luis F. Giraldo-Cadavid, Eduardo Tuta-Quintero, Jonathan Palacios, Stephanie Crevoisier, Diana C. Duarte, Marcela Poveda, Laura Cucunubo, Pablo Monedero

**Affiliations:** 1 Critical Care and Lung transplantation Service, Fundación Neumológica Colombiana, Fundación Cardio Infantil, Bogotá, Colombia; 2 Critical Care Service, Fundación Neumológica Colombiana, Fundación Cardio Infantil, Cra. 13b #161 – 85., Bogotá, 110131 Colombia; 3 Master’s Candidate in Epidemiology, Universidad de La Sabana, Chía, Colombia; 4 Facultad de Medicina, Universidad de La Sabana, Chía, Colombia; 5 Interventional Pulmonology Service, Fundación Neumológica Colombiana, Bogotá, Colombia; 6 Critical Care Service, Fundación Clínica Shaio, Bogotá, Colombia; 7 School of Medicine, Universidad de Navarra, Pamplona, España

**Keywords:** Airway extubation, Cough, Mechanical ventilation

## Abstract

**Background:**

Utilizing clinical tests, such as objective cough measurement, can assist in predicting the success of the weaning process in critically ill patients.

**Methods:**

A multicenter observational analytical study was conducted within a prospective cohort of patients recruited to participate in COBRE-US. We assessed the capability of objective cough measurement to predict the success of the spontaneous breathing trial (SBT) and extubation. Intra- and inter-observer reproducibility of the cough test and was evaluated using the intraclass correlation coefficient (ICC) and Cohen’s weighted kappa. We used receiver operating characteristic curves (ROC-curve) to evaluate the predictive ability of objective cough measurement.

**Results:**

We recruited 367 subjects who were receiving invasive mechanical ventilation. A total of 451 objective cough measurements and 456 SBTs were conducted. A significant association was found between objective cough measurement and successful SBT (OR: 1.68; 95% CI 1.48–1.90; *p* = 0.001). The predictive capability of the objective cough test for SBT success had a ROC-curve of 0.58 (95% CI: 0.56–0.61). Objective cough measurement to predict successful extubation had a ROC-curve of 0.61 (95% CI: 0.56–0.66). The intraobserver reproducibility exhibited an ICC of 0.94 (95% CI: 0.89–0.96; *p* < 0.001), while the interobserver reproducibility demonstrated an ICC of 0.72 (95% CI: 0.51–0.85; *p* < 0.001). The intraobserver agreement, assessed using Cohen’s weighted kappa was 0.94 (95% CI: 0.93–0.99; *p* < 0.001), whereas the interobserver agreement was 0.84 (95% CI: 0.67 − 0.10; *p* < 0.001).

**Conclusions:**

The objective measurement of cough using the method employed in our study demonstrates nearly perfect intra-observer reproducibility and agreement. However, its ability to predict success or failure in the weaning process is limited.

## Introduction

Invasive mechanical ventilation (IMV) plays a crucial role in managing critically ill patients in the intensive care unit (ICU) as it facilitates gas exchange, oxygenation, and carbon dioxide removal [1]. Once the acute or chronic respiratory failure that led to the need for IMV is resolved, ventilatory support is gradually withdrawn by conducting bedside trials predicting the success or failure of extubation [1–3]. Successful weaning process (WP) is defined as the transition to spontaneous ventilation without the need for reintubation within 48 h post-extubation [2]. However, failure in this process is common and can significantly increase complication rates, mortality, and healthcare costs. Therefore, clinical tests are needed to estimate the probability of success or failure in this process [1, 4, 5].

Currently, the respiratory conditions of patients undergoing IMV are assessed using predictive tests such as the rapid shallow breathing index, maximum inspiratory pressure (MIP), leak tests, diaphragmatic ultrasound measurements, and objective cough measurements to predict success in the spontaneous breathing trial (SBT) or extubation [6, 7]. Reports on cough evaluation in the literature mainly focus on measuring peak expiratory flow, requiring active patient collaboration and the use of specific technology to carry out the test [7, 8]. The assessment of involuntary cough strength constitutes a direct test of the ability to clear secretions from the tracheobronchial tree without requiring active patient collaboration or additional technological resources. Its application is characterized by simplicity, reliability, and reproducibility [8, 9]. Despite these qualities, it is important to note that complete validation of this test is still pending, limiting its clinical application in decision-making related to the withdrawal of mechanical ventilation [1, 6, 8, 10, 11].

Cough is a crucial physiological reflex in airway protection by facilitating secretion clearance [12, 13]. During orotracheal intubation, the vocal cords may not close properly, complicating the precise measurement of cough strength, a critical aspect for airway protection and secretion clearance [12, 13]. Observational studies have shown that weak cough is a predictor of extubation failure [14–16]. However, subjectivity in cough intensity assessment limits the generalization of these findings [7, 13, 14, 16].

The objective assessment of cough intensity is crucial for the success of the mechanical ventilation weaning process and extubation in critically ill patients [6–8, 10, 14, 15]. However, achieving this measurement is not straightforward, and despite numerous attempts at standardization and significant advances in clinical research regarding the appropriate timing for removing mechanical ventilation and extubation, limitations persist, hindering improvements in success rates in the ICU [6, 7, 10, 14, 16]. Therefore, in this study, we investigated the intra- and inter-observer reproducibility and reliability of objective cough measurement, as well as its ability to predict success in the successful SBT and extubation. These findings constitute secondary outcomes of the COBRE-US study [7].

## Methods

### Study period, location, and participants

A comprehensive observational prospective analytical study was undertaken at multiple centers within a prospective cohort of participants enrolled in the COBRE-US [7] study, spanning the period from February 2019 to November 2021. The study encompassed four intensive care units situated in high-complexity hospitals. All patients underwent a SBT, and the decision to extubate the patient was made based on the success of the trial, regardless of the cough evaluation results. Follow-up was conducted for 48 h post-extubation to determine its success [2, 6].

### Definition of the main objectives

The main objective of our study was to assess both intra- and inter-observer reproducibility or reliability of the objective cough measurement. Additionally, we aimed to describe the predictive capacity of this measurement in relation to successful SBT and extubation. A successful weaning process (successful SBT and extubation) leads to extubation with no need for ventilatory support for up to 48 h post-extubation [2, 7]. Conversely, the WP is deemed unsuccessful if the patient does not pass the SBT, necessitates reintubation within 48 h of extubation, or experiences mortality within the same timeframe [2, 7].

### Eligibility criteria

Inclusion criteria encompassed the absence of abundant respiratory secretions, resolution of the acute phase of the disease that necessitated mechanical ventilation, adequate gas exchange (or improvement compared to admission), stable cardiovascular condition (heart rate < 140 bpm, systolic blood pressure 90–160 mmHg, no vasopressor support or minimal support, norepinephrine < 0.1 mcg/kg/min, dopamine < 5 mcg/kg/min or inotropes, dobutamine < 5 mcg/kg/min), adequate metabolic state (pH in the range of > 7.35 to < 7.48, and normal levels of phosphorus, sodium, and potassium electrolytes), temperature ≤ 38 °C, hemoglobin ≥ 7 g/dl (≥ 10 g/dl in patients with coronary disease), Glasgow Coma Scale score ≥ 12, adequate oxygenation (SaO2 > 90% with FiO2 ≤ 0.4, PaO2/FiO2 > 150 mmHg, PEEP < 8 cm H2O). Pregnant patients, those with neuropsychiatric diseases, presence of diaphragmatic paralysis, and those who did not provide informed consent were excluded. The presence of delirium was not considered an exclusion criterion.

### Clinical variables

Data collection, including sex, age, weight, height, body mass index, presence of active smoking or alcoholism, and type of ventilatory failure, was obtained upon the patient’s admission to the ICU. The ICU therapist conducted an objective cough measurement after completing the SBT. These therapists received standardized training to perform this measurement and were not informed about the successful or unsuccessful outcome of the SBT. Two milliliters of normal saline solution (0.9%) were instilled through the closed suction catheter port at the end of inspiration. A cough strength rating method was implemented, utilizing a scale ranging from 0 to 3 Table 1. This scale ranged from no cough response to vigorous cough with expulsion of phlegm from the end of the endotracheal tube. This rating method showed a moderate correlation (*r* = 0.451, *p* < 0.001) with involuntary cough peak flow, as reported by Su et al. [10]. This objective cough evaluation has been replicated in subsequent studies [7].

**Table 1:** Objective measurement of cough

Rating	Description
0	No presence of cough
1	Audible movement of air through the orotracheal tube, but no audible cough
2	Strong cough with mobilization of secretions into the orotracheal tube
3	Strong cough with movement of secretions out (expulsion) of the orotracheal tube

All patients underwent a 30-minute SBT, either using a T-piece or pressure support ventilation. The determination of trial failure was based on the fulfillment of at least one of the following criteria: a partial pressure of arterial oxygen (PaO2) ≤ 60 mmHg or arterial blood oxygen saturation (SpO2) ≤ 90% with a fraction of inspired oxygen (FiO2) ≥ 0.50, PaCO2 > 50 mmHg or an increase > 8 mmHg from baseline, pH < 7.32 or a decrease > 0.7 units, respiratory rate ≥ 35/min or an increase ≥ 50% from baseline, heart rate ≥ 140 lpm or an increase ≥ 20% from baseline, systolic arterial pressure > 180 mmHg or an increase ≥ 20%, or systolic arterial pressure < 90 mmHg. The development of de novo cardiac arrhythmias, abrupt changes in mental status, and the presence of two or more indicators of respiratory distress, such as tachycardia, bradycardia, increased breathing effort, use of accessory muscles, abdominal paradox, facial signs of distress, diaphoresis, cyanosis, and marked dyspnea, were considered reasons to establish SBT failure [2, 7].

### Sample size

The reproducibility of objective cough measurement was determined by video recording 39 cough measurement tests. The calculated sample size was 34 patients for an expected intraclass correlation coefficient of 0.7 and a 95% confidence interval width of 0.35 [17, 18]. A total of 451 objective cough measurements were conducted [7]. Video tests were randomized and repeatedly assessed by the same observer to determine intra-observer reproducibility. Additionally, a second observer, unaware of the first observation’s result, evaluated the videos to determine inter-observer reproducibility.

### Statistical analysis

Data were transcribed into the Research Electronic Data Capture (REDCap) software [19]. All analyses were conducted using Stata version 16 (StataCorp LLC, College Station, USA). Continuous variables are presented as means and standard deviations (SD) or medians and interquartile ranges (IQR) based on their distribution, while categorical variables are presented as absolute and relative frequencies. The distribution of continuous variables was assessed using the Shapiro-Wilk test. The t-test or Mann-Whitney U test was employed for normally or non-normally distributed variables, respectively. Categorical variables were compared using the χ^2 test or Fisher’s exact test, as appropriate, based on contingency table frequencies.

Intra- and inter-observer reproducibility of the cough test was assessed using the intraclass correlation coefficient (ICC) and Cohen’s weighted kappa [17, 20]. We calculated the association between cough strength and duration of ventilation using the Spearman correlation coefficient. An ICC of 0.01 suggests “poor” agreement. ICC values ranging from 0.01 to 0.20 indicate “slight” agreement, those from 0.21 to 0.40 signify “fair” agreement, while ICC values in the range of 0.41 to 0.60 point to “moderate” agreement. When the ICC falls within the range of 0.61 to 0.80, the agreement is considered “substantial.” An ICC between 0.81 and 1.00 reflects an “almost perfect” level of agreement [17, 18]. Differences in cough measurement ratings based on patient age were evaluated using the Kruskal-Walli’s test. We assessed the discriminative ability of the objective cough measurement for predicting successful SBT and extubation using the C-statistic via receiver operating characteristic (ROC) curve analysis [18].

## Results

### Population characteristics

A total of 367 patients were included, with a median age of 61 years (SD: 49–72), and 57.9% (219/367) were males Table 2. The type of ventilatory failure in our population was hypoxemic in 75% (261/367), shock in 14.9% (52/367), hypercapnia in 6.6% (23/367), perioperative in 2.9% (10/367), and neuromuscular in 0.6% (2/367). The most common comorbidities were hypertension in 47.1% (173/367), diabetes mellitus in 30.8% (113/367), and chronic kidney disease in 18.8% (69/367).

**Table 2:** Population characteristics

Number of patients	367 (100)
man n (%)	219 (59.7)
Age, me(RIC)	61 (49–72)
Weight, me(IQR)	70 (60–80)
Height, m(SD)	163.6 (10)
Median Body Mass Index (IQR)	25.3 (21.7–29.1)
active smoking, n(%)	33 (9)
active alcoholism, n(%)	22 (6)
Diabetes mellitus n (%) arterial hypertension n (%) asthma n (%) pulmonary fibrosis n (%) chronic kidney disease n (%) chronic liver disease n (%)	113 (30.8) 173 (47.1) 8 (2.2) 6 (1.6) 69 (18.8) 17 (4.6)

Notes: n: number; me: medium; IQR: interquartile range; m: average; SD: standard deviation

### Objective cough measurement

A total of 451 objective cough measurement tests were conducted, with 57.6% of the tests resulting in a strong cough with secretion mobilization outside the endotracheal tube. Out of 367 patients, 346 (94.3%) were successfully extubated, and 31 patients (9%) were reintubated before 48 h Table 3.

**Table 3:** Features of objective cough measurement

Objective measurement of cough, *n*(%)*	
0 1 2 3	6 (1.3) 34 (7.6) 151 (33.5) 260 (57.6)
Successful extubation, n(%)	346/367 (94.3)
Extubation failures, n(%)	31/346 (9)

**Notas**: n: number

*451 objective cough measurements were performed

### Predictive capability of Objective Cough Measurement

**Fig. 1:**
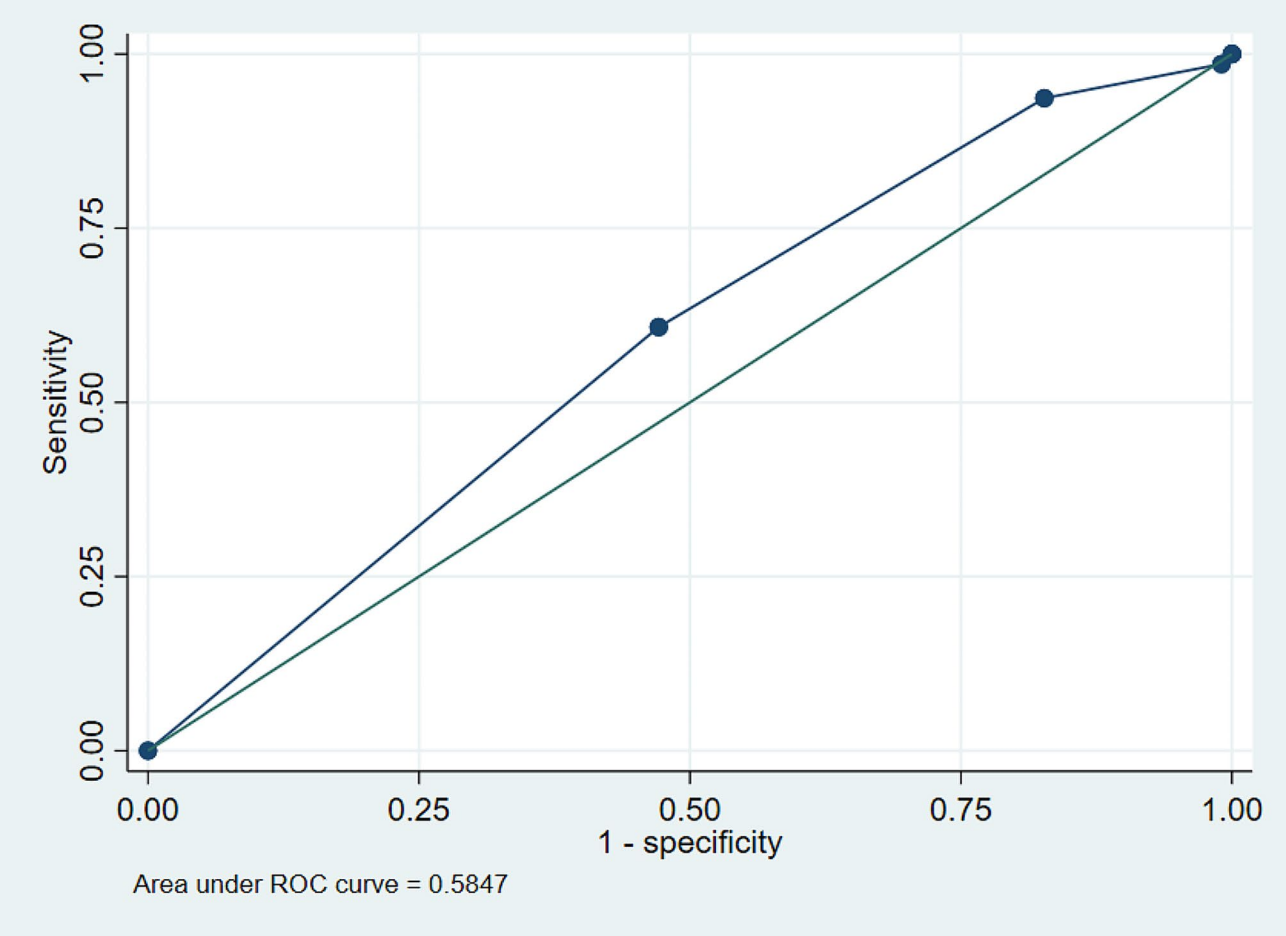
ROC-curve of the objective cough test for spontaneous breathing trial success

**Fig. 2:**
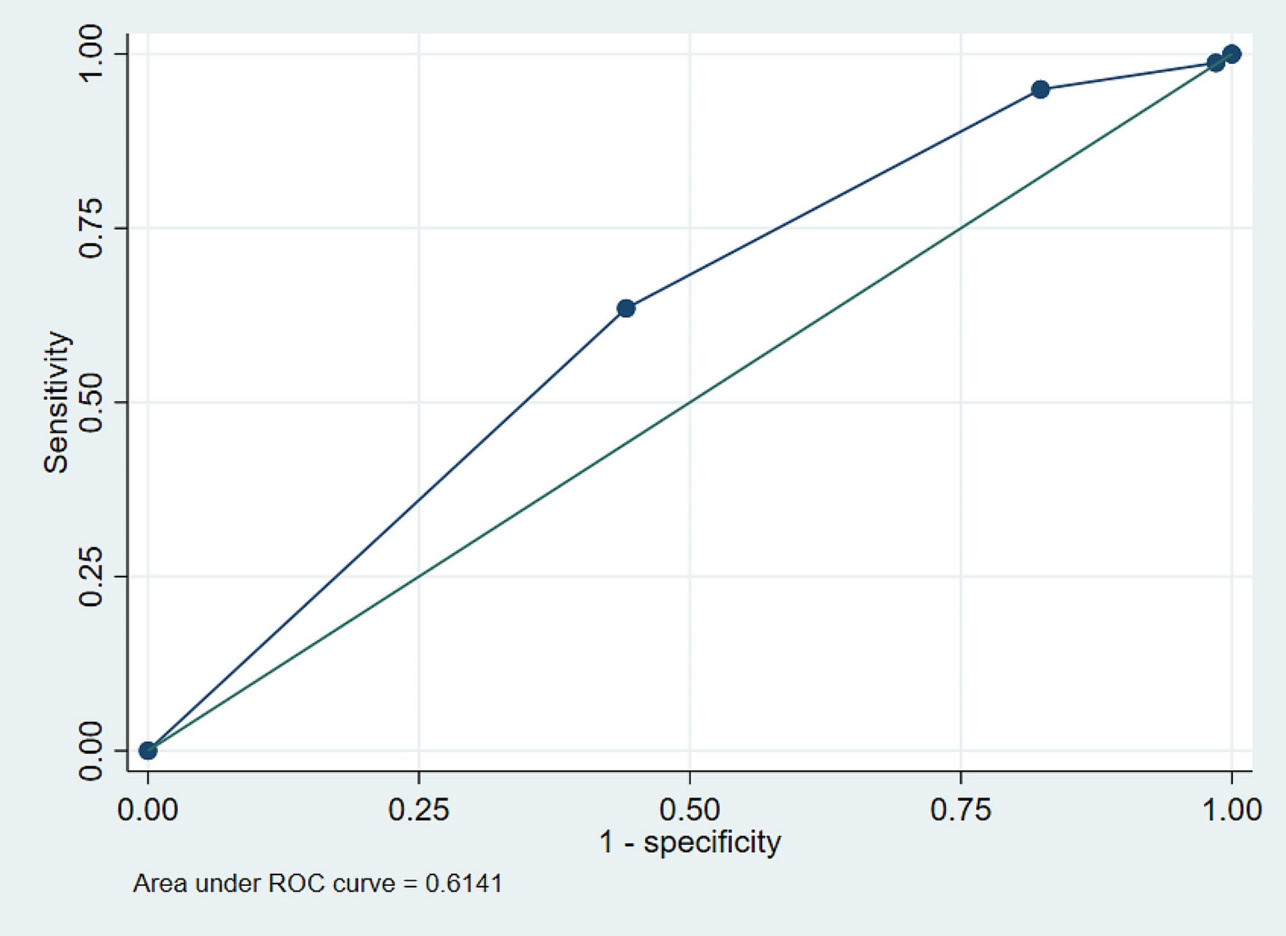
ROC-curve of the objective cough test for successful mechanical ventilation withdrawal

A significant association was found between objective cough measurement and successful SBT (OR: 1.68; 95% CI 1.48–1.90; *p* = 0.001). The predictive capability of the objective cough test for SBT success had a ROC-curve of 0.58 (95% CI: 0.56–0.61), Youden Index > = 3, sensitivity of 60.58%, specificity of 53.65%, a positive likelihood ratio of 13.072, and a negative likelihood ratio of 0.7346 Fig. 1. Objective cough measurement to predict successful extubation had a ROC-curve of 0.61 (95% CI: 0.56–0.66), Youden Index > = 3, sensitivity of 63.49%, specificity of 55.88%, a positive likelihood ratio of 14.39, and a negative likelihood ratio of 0.65 Fig. 2.

### Reproducibility or reliability of the objective cough measurement

**Fig. 3:**
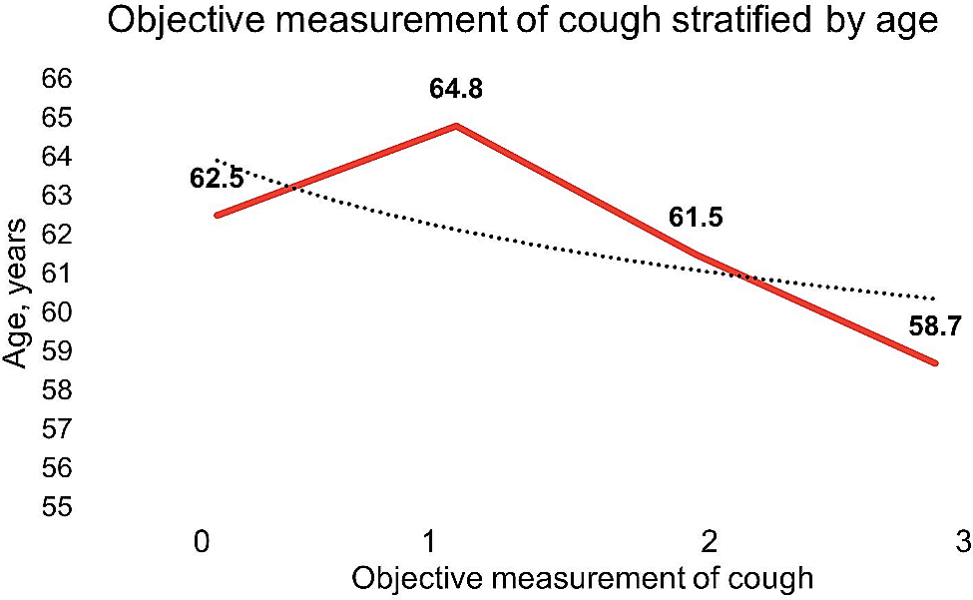
Age-stratified of objective cough measurement

The intraobserver reproducibility exhibited an ICC of 0.94 (95% CI: 0.89–0.96; *p* < 0.001), while the interobserver reproducibility demonstrated an ICC of 0.72 (95% CI: 0.51–0.85; *p* < 0.001). The intraobserver agreement, assessed using Cohen’s weighted kappa was 0.94 (95% CI: 0.93–0.99; *p* < 0.001), whereas the interobserver agreement was 0.84 (95% CI: 0.67 − 0.10; *p* < 0.001). The Spearman’s correlation coefficient between cough strength and duration of ventilation was 0.08 (*p* = 0.126). Age-stratified objective cough measurement is described in Fig. 3.

## Discussion

This study contributes to existing medical evidence regarding the clinical utility of objective cough measurement in patients undergoing IMV, highlighting almost perfect intraobserver reproducibility, while interobserver reproducibility showed substantial agreement. Cohen’s weighted kappa demonstrated nearly perfect agreement for both intraobserver and interobserver measurements, considering the maximum value in the test is three. The Spearman’s correlation coefficient revealed a very weak positive correlation between cough strength and duration of ventilation. Objective cough measurement exhibits a low predictive capacity for the success of the SBT and extubation. Therefore, further refinement and validation of cough measurement are required to optimize its clinical utility.

Our method of objective cough measurement, stratified ordinally considering the absence of cough and secretion mobilization, could be useful for predicting weaning in conjunction with clinical tests such as cuff leak test, SBT, rapid shallow breathing index, and diaphragmatic contraction velocity [12–14, 21, 22]. Furthermore, quantifying eliminated secretions could serve as a more natural method to estimate cough strength and predict various extubation outcomes in critically ill patients. This could be performed simultaneously with clinical tests such as the SBT, which are used to assess the success of the weaning process [21, 22].

Cough is a physiological response triggered voluntarily or involuntarily in response to irritation of the respiratory pathways with the goal of protecting the airway and imposing a demand on the function of respiratory muscles [23, 24]. A robust cough reflex allows the patient to properly expel airway secretions once extubated, preventing them from obstructing proper ventilation [7, 17, 25]. Additionally, a strong cough reflex implies proper functioning of inspiratory and expiratory nerves and muscles, increasing the likelihood of spontaneous breathing [25]. Therefore, an accurate measurement of this reflex should provide valuable information about the patient’s ability to manage secretions and mobilize an adequate volume of air after extubation. Consequently, it should serve to discriminate between patients who will succeed and those who will not in the mechanical ventilation withdrawal process [23–26].

Varón-Vega et al. [7] identified a significant relationship between objective cough measurement (OR: 1.90; 95% CI: 1.43–2.54; *p* < 0.001) and diaphragmatic contraction velocity (OR: 0.85; 95% CI: 0.73–0.99; *p* = 0.04) with extubation success in the multivariate logistic regression analysis. Additionally, they developed two predictive models using objective cough measurement: one for the success of the spontaneous breathing trial (0.56 × cough) – (0.13 × diaphragmatic contraction velocity) + 0.25, and another for extubation success (5.7 × spontaneous breathing trial) + (0.75 × cough) – (0.25 × diaphragmatic contraction velocity) – 4.5). The latter showed an area under the ROC-curve of 0.91 (95% CI: 0.87–0.95; *p* < 0.001). Our current results support the utility and reproducibility, both intra- and inter-observer, of objective cough measurement in clinical practice to identify patients prone to extubation success. However, it is crucial to emphasize that the combination of the SBT and objective cough measurement could enhance predictive performance and optimize clinical utility [7, 26].

In the study conducted by Su et al. [10], the effectiveness of maximum involuntary cough flow was examined as a predictor of extubation success in a sample of 150 patients, among whom 21.3% experienced extubation failure. Multivariate analysis revealed that a lower maximum involuntary cough flow was significantly associated with an increased risk of extubation failure (OR = 0.95; 95% CI: 0.93–0.98). Additionally, a threshold of the ROC-curve was identified as 58.5 l/min, with a sensitivity of 78.8% and specificity of 78.1% for predicting extubation success. Maximum involuntary cough flow is considered a metric associated with the ability to expel secretions and, according to our objective cough measurement method, would demonstrate a score ≥ 2 [10, 21]. Both the measure proposed by Su et al., and ours are non-invasive and could play a role as predictors of extubation success, especially when using other strategies such as the SBT [10].

Khamiees et al. [14] assessed the predictive capacity of extubation success by evaluating cough strength and the amount of endotracheal secretions. Cough strength was measured using a semi-objective scale ranging from 0 to 5, while the amount of endotracheal secretions was categorized as none, mild, moderate, or copious by a single observer. Patients coughed onto a white card placed 1–2 cm from the endotracheal tube, and if secretions impacted and wetted the card, it was considered a positive result for the white card test. Patients with weak cough (grades 0 to 2) were found to have four times higher odds of extubation failure compared to those with moderate to strong cough (grades 3 to 5). Similarly, those with moderate to copious secretions had over eight times higher odds of extubation failure than those with mild or absent secretions. Although objective measurement of cough and secretion expulsion from the endotracheal tube is useful for facilitating extubation decisions, it is rarely standardized due to subjectivity and lack of standardization in the ICU [7, 13–16]. Therefore, our results demonstrate an objective measurement of cough that can be successfully standardized, ensuring its validity and reproducibility among observers [21, 22].

Our results provide evidence regarding the optimal timing for extubating patients, emphasizing the importance of collaboration among a multidisciplinary team with expertise and training in conducting bedside tests such as diaphragmatic contraction velocity, SBT, leak test, and objective cough measurement [21, 22]. The latter measurement would enable the establishment of a consistent and reliable approach to measurements, both intra- and inter-observer.

### Limitations and strengths

Our research has certain limitations, such as the adoption of an observational methodology with data collection from clinical records. However, the research team possesses expertise in the interpretation, extraction, and appropriate synthesis of this type of data. The strengths of this study lie in its prospective and multicenter design, as well as the validation and reproducibility of objective cough measurement. While the subjective measurement of cough may pose a limitation, its good reproducibility, and the advantage of its simplicity, combined with the direct ability to measure secretion elimination, compensate for this aspect. The need for nasotracheal suctioning of patients after extubation, which is directly related to the effectiveness of cough, was not an objective of our study and therefore was not collected as data. Additionally, the lack of differentiation between types of ventilators weaning (easy, difficult, and prolonged) and variability in disease severity could impact the performance of objective cough measurement as a prognostic tool [7, 10].

## Conclusions

The objective measurement of cough using the method employed in our study demonstrates nearly perfect intra-observer reproducibility and agreement. However, its ability to predict success or failure in the weaning process is limited. It is suggested that the objective measurement of cough not be used as the sole test to determine extubation but rather as a complement to the SBT.

## Data Availability

The datasets generated during and/or analyzed during the current study are available from the corresponding author on reasonable request.
